# Complete Genome Sequence of a Sulfur-Oxidizing Bacterium, Alcaligenes faecalis Strain NLF5-7, Isolated from Livestock Wastewater

**DOI:** 10.1128/mra.00999-22

**Published:** 2023-01-04

**Authors:** Kook-Il Han, Sang Eun Jeong, Young Ho Nam, Hyun Mi Jin, Jong-Guk Kim, Mi-Hwa Lee

**Affiliations:** a Microbial Research Department, Nakdonggang National Institute of Biological Resources (NNIBR), Sangju-si, Gyeongsangbuk-do, Republic of Korea; b School of Applied Biosciences, Kyungpook National University, Daegu, Republic of Korea; University of Delaware College of Engineering

## Abstract

The complete genome sequence of Alcaligenes faecalis strain NLF5-7, which was isolated from livestock wastewater, is reported. The genome of strain NLF5-7 contains genes for assimilatory sulfate reduction, dissimilatory sulfate reduction and oxidation, and an SOX system, based on its functional genetic characteristics.

## ANNOUNCEMENT

Odors are emitted from various industrial areas, such as animal farms, wastewater treatment plants, paint shops, and various chemical industries (1). The major odors from wastewater treatment plants are sulfur compounds (hydrogen sulfide, methyl mercaptan) and nitrogen compounds (ammonia, trimethylamine) (2). Sulfide-oxidizing bacteria are widely applied in industry to convert toxic hydrogen sulfide into elemental sulfur (3). Recently, strain NLF5-7 was isolated during an investigation of odor-reducing bacteria.

Members of the species Alcaligenes faecalis subsp. *phenolicus* are Gram-negative, oxidase- and catalase-negative, and rod-shaped bacteria (4). Here, we describe the complete genome sequence and annotation of Alcaligenes faecalis strain NLF5-7, which was isolated from a livestock wastewater treatment plant in Nonsan, Republic of Korea. The livestock wastewater sample was serially diluted in saline (0.85% sodium chloride) and cultured on agar for sulfur-oxidizing bacteria (5) for 5 days at 30°C under aerobic conditions. Single colonies were then selected and continuously subcultured on fresh tryptic soy agar (Difco, USA) to obtain pure cultures. 16S rRNA gene sequencing was carried out using the universal primers 785F and 907R.

Strain NLF5-7 was grown in tryptic soy agar (Difco) for 3 days at 30°C. Colonies were then transferred using inoculation loops and suspended in the buffer supplied in the Wizard genomic DNA purification kit (Promega, USA) to isolate genomic DNA, as described in the manufacturer’s protocols. For long-read sequencing, DNA was sheared using a g-TUBE device (Covaris, USA), and a library was constructed using the 15-kb SMRTbell template preparation kit (Pacific Biosciences, USA). A size selection cutoff of 7 kb was used. The size-selected SMRTbell library was annealed and bound according to the SMRT Link setup and sequenced on a Sequel II instrument. To correct errors in the PacBio-assembled contigs, Illumina sequencing was also performed. A short-read sequencing library was constructed using the TruSeq Nano DNA library prep kit (Illumina, USA), followed by sequencing on an Illumina NovaSeq platform, with 151-bp paired-end reads. The library preparation and sequencing on both platforms were performed by Theragen Bio Institute (Republic of Korea). The 103,819 total reads (read *N*_50_, 11,022 bp) produced using the PacBio RS II platform were assembled; sequencing on the Illumina platform produced 10,470,802 total reads.

The long reads (PacBio) were *de novo* assembled using HGAP4 and Canu v1.7 software (6). Low-quality and short reads, adapter sequences, and terminal bases were filtered using Trimmomatic v0.38 (7), followed by alignment against the PacBio circular contig using BWA-MEM v0.7.17 (8). The final error correction was conducted five times using Pilon v1.22 (9), with a default minDepth value of 0.01, resulting in a single circular 4.43-Mbp chromosome (Table 1). Using PGAP v6.1 (10) for genome annotation, 4,015 protein-coding genes, 9 rRNA genes, 57 tRNA genes, 4 noncoding RNA genes, and 32 pseudogenes were predicted. Default parameters were used except where otherwise noted.

**TABLE 1:** Summary of the assembly and annotation statistics for *A*. *faecalis* NLF5-7

Parameter	Data
Genetic element	Chromosome
Length (bp)	4,431,216
GC content (%)	55.48
No. of coding sequences	4,015
No. of rRNAs	9
No. of tRNAs	57
Sequencing depth (×)	199
GenBank accession no.	CP100755

We found that various genes involved in sulfate metabolism were identified in the genome. A gene cluster containing an assimilatory sulfate reduction (*sat*, *cys*ND, *cys*H, *cys*JI), dissimilatory sulfate reduction and oxidation (*sat*), and SOX system (*sox*Y, *sox*Z) was present.

### Data availability.

The genome sequence and raw sequencing reads for strain NLF5-7 were deposited under GenBank accession number CP100755, BioProject accession number PRJNA807446, BioSample accession number SAMN25982028, and Sequence Read Archive (SRA) accession numbers SRX18083056 and SRX18083055. Alcaligenes faecalis strain NLF5-7 has been deposited in the Korean Collection for Type Cultures under accession number KCTC 14797BP.
